# Letter to the editor: healthy alternatives to *trans *fats

**DOI:** 10.1186/1743-7075-4-10

**Published:** 2007-04-27

**Authors:** Frédéric Destaillats, Julie Moulin, Jean-Baptiste Bezelgues

**Affiliations:** 1 Nestlé Research Center, Lausanne, Switzerland

## Abstract

Consumption of *trans *fats is associated with an increase of cardiovascular disease (CVD) risk. To comply with regulatory policies and public health authorities recommendations, *trans *fats should be replaced in food products. The study by Sundram *et al.* (Nutrition & Metabolism 2007, 4:3) reporting the effect on CVD risk factors of interesterified fat (IE) and partially hydrogenated soybean oil (PHSO) compared to palm olein (POL) has been critically analyzed. The study design and in particular the composition of the tested fats was not suitable to properly answer the question raised regarding the effect of alternative ingredients to *trans *fats on plasma lipids. The observed effects are divergent with predicted data derived from the literature model consolidated using the individual results of 60 randomized clinical trials. The results of the study published by Sundram and co-workers have to be considered with awareness.

## 

Dear Editor,

Diet and health is now recognized to be a critical factor in causing diseases of metabolic origin. Fats in particular are documented to influence the risk of various degenerative diseases. Consumption of monounsaturated *trans *fatty acids (TFA) has been shown to be associated with increased risk of cardiovascular disease (CVD) [1,2]. Most public health agencies have implemented labeling or ingredient restrictions on *trans *fats. Alternative ingredients to *trans *fats are now available in the food supply allowing the reformulation of food products that is mandatory to comply with policies and advices from health authorities. The physical properties and the chemical stability of *trans *fats were the two major reasons that explained the popularity of this ingredient. The reformulation of food products is difficult because the intrinsic characteristics of the products should be maintained.

Ingredients such as palm olein (POL) produced by physical fractionation of palm fat or interesterified (IE) fats produced from fully hydrogenated vegetable oils could be used as alternative ingredients to *trans *fats in food products. The major saturated fatty acids present in these fats are palmitic and stearic acids. These two fats are produced using industrial processes flexible enough to obtain the desire physical properties. In a recent paper published in The Journal [3], Sundram and co-workers reported on the effect on CVD risk factors of partially hydrogenated soybean oil (PHSO), IE and POL. The main comparison was IE and PHSO versus POL. The composition and the physical properties of the three fats compared were not the same. The melting points of the IE and PHSO fats were about 15 to 20°C higher that POL, leading to different applications in the food industry. As a matter of fact, the relative distribution between saturated, monounsaturated and polyunsaturated fatty acids were different for all the fats evaluated. In particular, the content of saturated fatty acid was about 30% higher in the IE fat than in the POL fats; and the content in monounsaturated fatty acids, 2.3 times lower in IE than in POL.

In the frame of the on-going efforts aiming at replacing *trans *fats in food products, a pertinent strategy would have been to compare, in a double blind fashion, the effects on CVD risk of food items produced from three fats having the same physical properties (*i.e. *identical melting points). The primary objective (the hypothesis) would have been to compare the nutritional effects of POL and IE to PHSO in order to identify the healthy alternative to *trans *fat (PHSO) in a controlled design. The experimental conditions used by the investigators were not suitable to answer the question. Indeed, changes in CVD risk factors such as low-density lipoprotein cholesterol (LDL-C), high-density lipoprotein cholesterol (HDL-C), total cholesterol and diagnostic ratios were expected from literature data.

It is possible to predict the effects, on plasma lipids, of fats and oils using mathematical models obtained recently, as a result of a large metaanalysis conducted by Mensink and co-workers [2]. The predicted and reported effects of the fats evaluated in the study from Sundram and co-workers [3] are given in the Figure 1. Confidence intervals (CI) were constructed using metaanalysis coefficients confidence intervals [2]. They represent an estimation of what should be CI and they help defining the credibility of some study results according to metaanalysis results. The effect on HDL-C of IE and PHSO compared to POL measured in the clinical trial compared with the expected effect predicted by the literature model (see Figure 1A). However, the effects on total cholesterol (see Figure 1B), on LDL-C (see Figure 1C) and on the diagnostic ratio total cholesterol to HDL-C (see Figure 1D) differ from the prediction.

**Figure 1:**
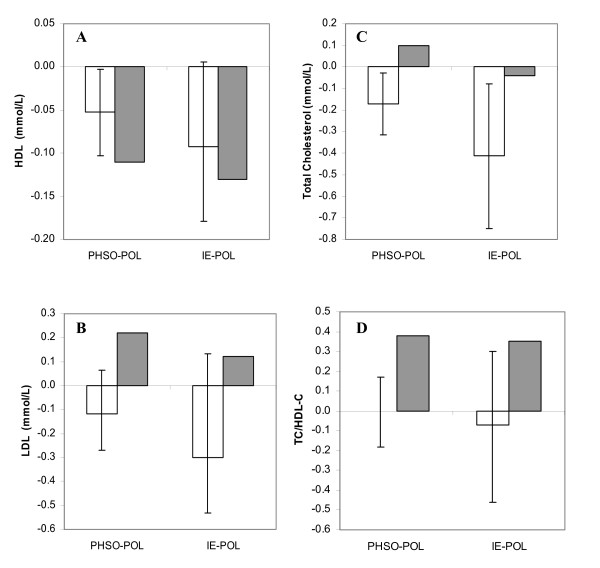
Predicted (open bars) and measured (grey bars) changes in HDL-C (**A**), total cholesterol (**B**), LDL-C (**C**), and total cholesterol to HDL-C ratio (**D**) when consumption of partially hydrogenated soybean oil (PHSO) or interesterified (IE) fat is compared to palm olein (POL). Measured data are adapted from the study of Sundram and co-workers (3). Estimation of predicted effects of the PHSO and IE compared to POL have been calculated using the equations proposed by Mensink and co-workers (2) and the fatty acid composition of the fats used in the study from Sundram and co-workers (3)

Sundram and co-workers [3] hypothesized that the unexpected effects of IE on the monitored CVD risk factors could be due to the regiospecific distribution of stearic acid on the triacylglycerols (TAG) formed by chemically-catalyzed interesterification. The goal of the interesterification process is to change the physical properties of the starting material: a blend of fats and oils. The melting point of the interesterified fat is usually lower. The analytical method used by Sundram and co-workers [3] to analyze the test fats is widely used to determine the TAG profile of edible fats and oils. However, this method is not suitable to assess the regiospecific distribution of fatty acids or the molecular species of TAG. Two TAG regio-isomers have the same fatty acids esterified in the *Sn*-glycerol but not at the same position [*e.g. *1,2-dipalmitoyl-3-oleoyl-glycerol (PPO), and 1,3-dipalmitoyl-2-oleoyl-glycerol (POP)]. Reversed-phase high-performance liquid chromatography (RP-HPLC) analysis of TAG cannot discriminate TAG regioisomers. The difference between PPO and POP is that the oleic acid is esterified in *Sn*-1(3) or *Sn*-2 position on the *Sn*-glycerol, respectively. These structural data could be assessed by either partial deacylation of TAG using pancreatic lipase or Grignard reagent [4], by mass-spectrometry [5] or by NMR [6].

The neutral effect of stearic acid, compared to oleic acid, on CVD risk factors such as HDL-C, LDL-C and diagnostic ratio has been recently confirmed by Thijssen and Mensink [7]. These recent data are consistent with studies showing that stearic acid compared to oleic acid does not alter plasma lipids [8-10]. The results obtained by Sundram and co-workers [3] are divergent with the predicted data, derived from the literature model consolidated using the results of 60 randomized clinical trials. The design of the study was not suitable to challenge the hypothesis raised by the investigators. Alternatives to replace *trans* fats in food products are available. However, the study published by Sundram and co-workers does not allow concluding that palm oil is healthier than interesterified fats.

## Abbreviations

Cardiovascular disease (CVD); Confidence intervals (CI); High-density lipoprotein cholesterol (HDL-C); Interesterified fats (IE); Low-density lipoprotein cholesterol (LDL-C); Nuclear magnetic spectroscopy (NMR); Palm olein (POL); Partially hydrogenated soybean oil (PHSO); Triacylglycerols (TAG)

## Competing interests

The authors declare that they have no competing interests.

## Authors' contributions

Frédéric Destaillats, Julie Moulin and Jean-Baptiste Bezelgues participated equally to the critical analysis of the study published by Sundram and co-workers (3).
